# Impaired Geotaxis as a Novel Phenotype of Nora Virus Infection of *Drosophila melanogaster*

**DOI:** 10.1155/2020/1804510

**Published:** 2020-07-18

**Authors:** Abigail Rogers, Lesley Towery, Amanda McCown, Kimberly A. Carlson

**Affiliations:** Department of Biology, University of Nebraska, Kearney, NE 68849, USA

## Abstract

Nora virus (NV) is a picorna-like virus that contains a positive-sense, single-stranded RNA genome. The virus infects *Drosophila melanogaster* with no known characterized phenotype. In this study, geotaxis assays and longevity analyses were used to determine if Nora virus infection affects *D. melanogaster*'*s* locomotor ability. In addition, Drosophila C virus (DCV), a well-characterized *D. melanogaster* virus, was used as a positive control, as it has previously shown a locomotor defect in infected flies. Stocks infected with NV (NV+) and DCV (DCV+) and virus-free (NV-/DCV-) stocks were established. Over a 3-year period, approximately 2,500 virgin female flies were tested for geotaxis and longevity using Kaplan–Meier analyses, as well as the Cox Proportional Hazards regression for survivorship. There was a significant decrease in the geotaxis when the *D. melanogaster* flies were infected with Nora virus compared to uninfected controls, but no difference was found between DCV+ and NV+ trials. There were not significant differences in longevity between the three groups. This is the first time that a phenotype has been recorded in association with Nora virus infection. Overall, the data demonstrate that geotaxis dysfunction may be a phenotypic hallmark of Nora virus infection.

## 1. Introduction

*Drosophila melanogaster* is a model organism commonly used in biological studies to study virus effects because its DNA is similar enough to humans that they can be used as a model for human immunity and disease [1], as well as development and behavior. One behavioral aspect similar between *D. melanogaster* and humans is geotaxis [2]. Geotaxis is a response to gravitational force and can be used to study the locomotor function of an organism. In *D. melanogaster*, geotaxis is an innate behavior that causes the fly to climb the wall of a cylinder after being tapped to the bottom [3]. By quantitating geotaxis, it was observed that Drosophila C virus (DCV) causes a decrease in locomotor activity in male flies [4]. Although locomotor function is a basic measurement, it is vital for other important behaviors such as survival and reproduction [5]. Another important characteristic to be studied in response to viral infection is longevity. The lifespan of *D. melanogaster* is comparatively short, which makes studying the effects of a virus on longevity relatively easy. *D. melanogaster* viruses have variable effects on longevity. There is genetic variation in survivorship with DCV infection, which is dependent upon a number of factors. One early study demonstrated that DCV infection caused a decrease in lifespan with 30–50% of the flies dying within a week [6]. While others, such as Rhabdovirus sigma, are not lethal and have no effect on lifespan [7]. Therefore, the importance of locomotor function and longevity in *D. melanogaster* makes these good metrics for characterizing a possible disease phenotype associated with a viral infection, especially one that has no known phenotype, such as Nora virus.

Nora virus is a picorna-like virus, similar to poliovirus, rhinoviruses, and enteroviruses, that replicates in the gut of *D. melanogaster* and is transmitted horizontally via the fecal-oral route [8]. The virus genome consists of a single-stranded, positive-sense genome and has four open reading frames (ORFs) that encode viral replication enzymes and capsid proteins [9]. Interestingly, *D. melanogaster* flies that are chronically infected with Nora virus as the primary innate immune response using siRNA showed no effect on Nora virus. This is unlike most viral infections, as this is active against foreign RNA and is essential in defense of most RNA viral infections [10]. In addition, there was no evidence that the virus had an effect on the overall fitness of *D. melanogaster* [8]. Therefore, currently no observable phenotype has been associated with Nora virus infection of *D. melanogaster.*

In this study, geotaxis assays and lifespan analysis were used to observe the effects of Nora virus infection on both locomotor function and longevity of *D. melanogaster.* For comparison, DCV, a well-characterized *D. melanogaster* virus, was used as a control because infected flies have been shown to have decreased locomotor function in terms of geotaxis and, in some studies, decreased longevity. We predicted that *D. melanogaster* infected with Nora virus will show reduced climbing ability and possibly a decrease in lifespan compared to virus-free flies.

## 2. Materials and Methods

### 2.1. *D. melanogaster* Husbandry and Viral Infection

Witi *Rel*^E23^ stocks (a kind gift from Dan Hultmark from Umeå, Sweden) were maintained at 25°C on standard cornmeal, molasses, and torula yeast medium with diurnal light. Flies were infected fecal-orally to establish either Nora virus infected or DCV infected stocks for further analysis [11]. Once adequate stocks were established, stock bottles were expanded for fly collection by transferring flies into new bottles. The next generation of flies was allowed to eclose, and virgin females were collected for further analysis. The uninfected flies were kept in a separate incubator in the insectarium room of UNK's Biology Department to prevent contamination. Infected flies were kept in a Nora virus positive incubator in a separate room.

### 2.2. Verification of Nora Virus and DCV Infection with RNA Extraction and RT-PCR Analysis

Total RNA extraction was performed using TRIzol® as per manufacturer's instructions (Thermo Fisher Scientific, Waltham, MA). Each sample was quantified using a NanoDrop™ 2000c spectrophotometer (Thermo Fisher Scientific) to assess RNA purity and concentration. Samples were analyzed for the presence of Nora virus using *NORF1 55–844* (Forward 5′-TGGTAGTACGCAGGTTGTGGGAAA-3′; Reverse 5′-AAGTGGCATGCTTGGCTTCTCAAC-3′) primers or DCV using *DCV7/8* (Forward 5′-AGTATGATTTTGATGCAGTTGAATCTC-3′; Reverse 5′-GAAGCACGATACTTCTTCCAAACC-3′) primers and Verso 1-Step RT-PCR Hot-Start Kit according to manufacturer's instructions (Life Technologies Inc., Carlsbad, CA). The positive controls were an RNA extraction that previously tested positive for Nora virus infection and DCV cDNA for DCV infection. Reactions were set up under the following conditions: Nora virus: 50°C for 30 min; 94°C for 2 min; 94°C for 30 s, 55°C for 30 s, and 68°C for 1 min for 30 cycles; 68°C for 5 min; and hold at 4°C. DCV: 50°C for 30 min; 94°C for 4 min; 94°C for 40 s, 52°C for 40 s, and 72°C for 1 min for 35 cycles; 72°C for 7 min; and hold at 4°C. Samples were analyzed on a 0.8% agarose gel in a TAE buffer solution at 80 V for 1 hour. A positive reaction yielded a product at approximately 790 bp for Nora virus and 524 bp for DCV.

### 2.3. Experimental Setup

Once stocks were confirmed to be either Nora virus or DCV infected or uninfected, bottles of Nora virus positive (NV+), Nora virus negative (NV-), and/or DCV positive (DCV+) stocks were established using 30 mating pairs in each and allowed to mate for 24 hours. After 24 hours of mating, the parental generation was transferred into new bottled medium and allowed to mate for another 24 hours. The F1 generation of virgin female flies were collected every 6 hours after first emergence. Experimental flies were kept in small cages, approximately a liter in volume (∼16 cm in total height), with ventilation and food vial extension tube. New vial food media were replaced every 3 days. Each cage contained 60 females, and at the end of 3 years there were 1140 NV+ females tested, 840 NV− females tested, and 600 DCV+ females tested. Overall, 20 cages were completed for NV+, 15 for NV−, and 10 for DCV+.

### 2.4. Geotaxis and Longevity Assays

The treatment groups were maintained in the liter sized cages for the entirety of their lifespan. Every 3 days, the dead were collected via aspiration and the number was tallied. This continued until all the flies died. Dead *D. melanogaster* were frozen at −80°C for future RNA analysis for the presence or absence of Nora virus, as well as quantification. After the dead were removed from the cage, the food was removed, and the hole was blocked with an empty food vial. Each cage was gently tapped on the counter to get all flies into the bottom of the cage. A timer was set for 1 minute and the flies were allowed to climb to the top. At the end of the 1 minute, the number of flies that climbed to the threshold line, which was 2/3 of the way to the top (10.5 cm), was counted and recorded. The empty food vial was then replaced with a new food vial. The data was collected for three years and sent to the Bioinformatics and Systems Biology Core at the University of Nebraska Medical Center (UNMC) for statistical analysis. For both geotaxis and longevity assays, the statistical significance between treatment groups was determined using Kaplan–Meier analysis. In addition, the longevity data was subjected to Cox Proportional Hazards regression analysis. Both tests were done with an alpha of 0.05.

### 2.5. RT-qPCR for Nora Virus

Total RNA was extracted as described in Section 2.2 for days 2, 21, and 40 to determine Nora virus load at an early, middle, and late age. TaqMan Gene Expression Assay kits (Applied Biosystems, Foster City, CA) and the 7500 Real Time PCR System (Applied Biosystems) were used to perform reverse transcription quantitative PCR (qRT-PCR) according to manufacturer's instructions. The TaqMan probe sets were *Ribosomal protein L32* (*RpL32*; endogenous control; assay #Dm02151827_g1) and *Nora virus* (AIRSA9W). Reactions were carried out in triplicate using the following conditions: 48°C for 30 min, 95°C for 10 min (95°C for 15 s, 60°C for 1 min) repeated for 40 cycles. PCR products were analyzed in the linear range for amplification using the 7500 Real Time PCR Sequence Detection System software and normalized to *RpL32*. The quantitative results were analyzed on a log 2 scale and with the ΔΔCT method to determine viral load [12]. Statistical analysis was conducted using an unequal variance, two-tail Student's *t*-test with an alpha of 0.05.

## 3. Results

### 3.1. Geotaxis Analysis

**Figure 1:**
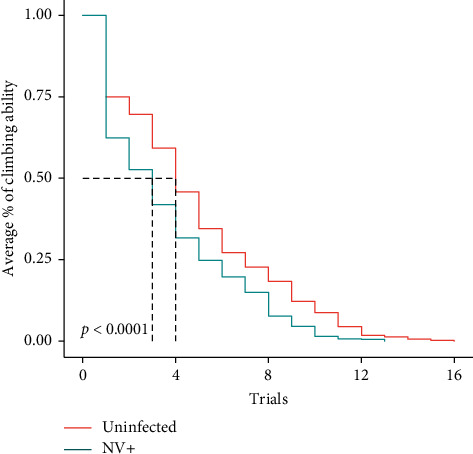
Analysis of geotaxis every 3 days in NV+ and uninfected females. Geotaxis was measured by counting the number of flies that climbed above the line after 1 minute and the average climbing ability calculated. Kaplan–Meier geotaxis analysis demonstrated a significant difference between the two groups (*p* < 0.0001) with uninfected flies having greater locomotor function. Trials are representative of the day of testing; for example, trial 4 occurred on day 12.

**Figure 2:**
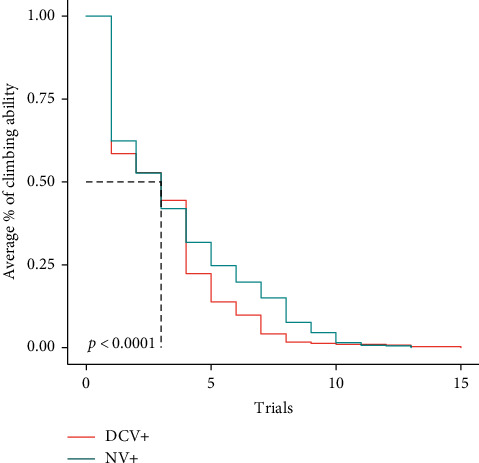
Analysis of geotaxis every 3 days in NV+ and DCV+ females. Geotaxis was measured by counting the number of flies that climbed above the line after 1 minute and the average climbing ability calculated. Kaplan–Meier geotaxis analysis demonstrated a significant difference between the two groups (*p* < 0.0001) with NV+ having greater locomotor function at older ages. Trials are representative of the day of testing; for example, trial 5 occurred on day 15.

The geotaxis analysis showed a significant difference in the locomotor function between NV+ and uninfected flies (*p* < 0.0001). The uninfected group had a greater climbing ability than the NV+ (Figure 1). When comparing NV+ to the DCV+, the data showed there was a significant difference in the climbing ability between the two groups (*p* < 0.0001) with the NV+ having greater locomotor function than the DCV+ (Figure 2). In DCV+ versus the uninfected control flies, the Kaplan–Meier geotaxis analysis showed that there was significant difference in the climbing ability between the two groups (*p* < 0.0001). The uninfected control flies had greater locomotor function than the DCV+ (data not shown).

### 3.2. Survivorship Analysis

**Figure 3:**
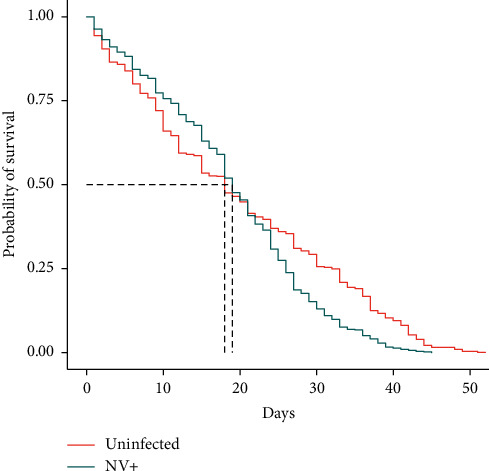
Survivorship analysis of Nora virus infected versus uninfected treatments. Kaplan–Meier survivorship analysis using a log-rank Mantel–Cox test demonstrated a significant difference between the two groups (*p* < 0.0001), but there was no overall significant effect of infection on survivorship as determined by the Cox Proportional Hazards regression analysis (*p*=0.9845).

**Figure 4:**
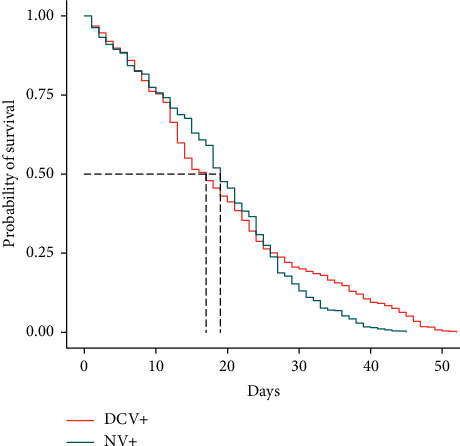
Survivorship analysis of Nora virus infected (NV+) versus Drosophila C virus infected (+) treatments. The Kaplan–Meier survivorship analysis using a log-rank Mantel–Cox test identified a significant difference between the two groups (*p*=0.00029), but there was no overall significant effect of infection type on survivorship as determined by the Cox Proportional Hazards regression analysis (*p*=0.9995).

The Kaplan–Meier survivorship analysis using a log-rank Mantel–Cox test showed an overall significant difference in the longevity between the three treatment groups (*p* < 0.0001). When comparing two treatment groups at a time, for NV+ versus uninfected, it was *p* < 0.0001 (Figure 3); for NV+ versus DCV+, it was *p*=0.00029 (Figure 4); and it was not significant for DCV+ versus uninfected (*p*=0.94; data not shown). When studying the survivorship curves, it is evident that they appear to cross each other at around 20 days of age (Figures 3 and 4). In addition, the median survival for the three treatment groups was relatively similar (uninfected = 18 days; NV+ = 19 days; DCV+ = 17 days). Therefore, Cox Proportional Hazards regression analysis was performed to determine if there was any significant effect of infection status on survivorship. The log-likelihood ratios were not found to be significantly different in any of the cases and the *p* values also demonstrated that there is no significant effect of infection on survivorship or longevity.

### 3.3. Validation of Nora Virus with RT-PCR and RT-qPCR

All Nora virus infected samples demonstrated the expected RT-PCR amplification product for Nora virus *ORF1* at 790 bp, whereas the uninfected controls did not (data not shown). Quantitative RT-PCR was used to analyze Nora viral load at three time points (days 2, 21, and 40). Average fold change in Nora virus load was determined relative to day 2 of infection. As infection progressed relative to day 2, the viral load fluctuated, decreasing at day 21 (1.5-fold decrease) and increasing again at day 40 (25.05-fold increase). The decrease between days 2 and 21 was not statistically different. The increase in Nora virus viral load at day 40 was statistically significant (*p* < 0.02) when compared to both days 2 and 21. The average fold change from days 21 to 40 was a 39.16-fold increase.

## 4. Discussion and Conclusions

The current study demonstrates a possible phenotype associated with Nora virus infection, which is a decrease in climbing ability (geotaxis). Nora virus is a picorna-like virus [9], which is similar to the picornavirus, human poliovirus. Before a viable vaccine, human poliovirus infection led to muscle weakness, locomotor disability, and sometimes complete paralysis [reviewed in [13]]. Geotaxis in *D. melanogaster* has been used to measure the pathogenicity of other human diseases, including Parkinson's disease [14, 15] and Alzheimer's disease [16]. Because of Nora virus' relatedness to human diseases in the picornavirus family, specifically poliovirus, we hypothesized that it is plausible that Nora virus infection may result in locomotor dysfunction in *D. melanogaster*. Indeed, the geotaxis analysis showed that there was a significant decline in the climbing ability of Nora virus infected flies as compared to the uninfected flies (Figure 1). This result indicates a potential phenotype associated with Nora virus infection. This finding is contrary to a study that suggested that there were no serious pathological effects on the overall fitness of the Nora virus infected flies [8], but this study never looked at geotaxis as a possible phenotype. The results also indicated a significant difference between Nora virus infected and DCV infected flies, with the DCV flies having decreased climbing ability (Figure 2). This is consistent with previous studies that indicated a decrease in locomotor function when *D. melanogaster* flies were infected with DCV [4, 5]. It also suggests that Nora virus may be slightly more infectious than DCV, but this needs to be tested. Overall, this data suggests that Nora virus is affecting the motor skills of *D. melanogaster,* but it is important to also determine the role of aging in this process. It is known that over time, *D. melanogaster* flies, as with many other organisms, have a degradation of their motor skills [3, 17].

To begin to understand this, we carried out longevity analyses in conjunction with the geotaxis assays. A previous study suggested that the survival rates were similar between Nora virus infected and uninfected flies. However, in that study, they did not perform the statistical analyses to determine if longevity was in fact impacted [8]. In this study, we encompassed the entire lifespan of *D. melanogaster*, recording deaths every three days in conjunction with the geotaxis assays. The Kaplan–Meier survivorship analysis using a log-rank Mantel–Cox test indicated a significant decline in longevity between the Nora virus infected and uninfected groups (Figure 3), as well as between DCV+ and NV+ (Figure 4), but no significant difference between DCV+ and uninfected flies. When comparing the survivorship curves, it was observed that the data intersected and crossed at approximately 20 days of age and that the three treatment groups had similar median survival ages (uninfected = 18 days; NV+ = 19 days; DCV+ = 17 days). Due to the disparity in the data, Cox Proportional Hazards regression analysis was performed and it determined that there was no significant effect of infection status on survivorship. When analyzing the survivorship curves and comparing NV+ to both uninfected (Figure 3) and DCV+ (Figure 4) flies, it appears that initially the uninfected and DCV+ groups declined more quickly and then stabilized to live longer overall, with some flies in the DCV+ and uninfected groups living as long as 52 days, whereas the NV+ only lived up to 45 days. This possible decline in survivorship in NV+ populations may be correlated to the significant increase in Nora virus load from day 21 to day 40. As the *D. melanogaster* flies age, they may accumulate NV and die more rapidly (Figure 3). Interestingly, Nora virus load across the lifespan demonstrates a biphasic model, which is seen in other RNA viruses like HIV [18] and influenza [19], as well in an earlier study done with Nora virus [20]. *In toto*, the data suggest that Nora virus infection may decrease the overall longevity of *D. melanogaster*, but this could be due to a number of factors including genetic variation and not only infection with Nora virus.

Overall, the data suggest that Nora virus infection leads to a decrease in geotaxis. The data demonstrates that Nora virus infection is similar to DCV infection with a decline in locomotor ability, and therefore the locomotor defect may result in the same way with both viruses. *D. melanogaster* flies have an open circulatory system, meaning that the hemolymph is pumped by the heart into the tissues and is diffused back to the heart. In the case of DCV, infected hemolymph is circulated to the brain, via the open circulatory system, where it establishes infection [21], and quite possibly results in the locomotor defect seen in infected flies. Studies are currently underway to determine if Nora virus is circulating in the hemolymph of infected flies. If it is, future studies will be done to determine if the virus is being transported to the brain and identify the effect on geotaxis. In addition, further characterization of locomotor activity will be done to determine if the defect is only a geotaxis issue or an overall disability in movement. Overall, the data presented is the first account of a potential phenotype, geotaxis defect, associated with Nora virus infection.

## Data Availability

The data related to this article are available from the corresponding author upon request.
